# New high throughput fully automated plasma p‐Tau217 immunoassay

**DOI:** 10.1002/alz.087951

**Published:** 2025-01-09

**Authors:** Miklos Szabo, Paul Wynveen, Chris Knutson, Mike Salvati, Katie Hoffmann, Kara Curtis, Ben Dumke, Jason Patzlaff, Mikaela Nichkova‐Doseva

**Affiliations:** ^1^ Beckman Coulter Inc., Chaska, MN USA

## Abstract

**Background:**

Hyperphosphorylated Tau is a pathologic hallmark of the neurofibrillary tangles in Alzheimer’s Disease (AD). p‐Tau_217_ is a promising blood‐based biomarker in AD research, drug development, diagnosis, disease monitoring, and patient care. Several assays for p‐Tau_217_ in plasma have been previously described, however there is a need to make this test easily available to the clinical laboratories around the world and more accessible to the patients and the clinicians. We describe the development of a precise and highly sensitive plasma phosphorylated Tau (Thr 217) high‐throughput immunoassay on the new Beckman Coulter DxI 9000^TM^ Access Immunoassay Analyzer.

**Method:**

The prototype p‐Tau_217_ assay is a two‐step sandwich assay using an alkaline phosphatase‐conjugated anti‐tau monoclonal antibody (MAb), with an anti‐p‐Tau217 MAb bound to paramagnetic particles. EDTA plasma sample, reaction buffer, and MAb‐coated particles are incubated and washed. This is followed by addition of the ALP‐conjugate with another incubation and wash step before a chemiluminescent substrate is added. The light generated is directly proportional to the p‐Tau_217_ concentration in the sample. Through the capabilities of the DxI 9000 analyzer, assay time to first results is ∼48 minutes with a throughput of 225 test/hour. For this study, 66 EDTA plasma samples, consisting of 40 AD and 26 age‐matched healthy controls, were evaluated. Analytical sensitivity (lower limit of quantitation) and discrimination between AD positive and healthy control populations were evaluated.

**Result:**

The prototype p‐Tau_217_ assay has a targeted analytical measuring range of ∼10 fg/ml to approximately 2000 fg/mL. All (100%) healthy control samples were quantified, and a 20% within‐run lower limit of quantitation (LLOQ) was estimated at 10 fg/mL with a 10% LLOQ estimated at 30 fg/mL. The ratio of the mean concentration of p‐Tau_217_ in the AD samples (520.9 fg/mL) compared with the mean concentration of p‐Tau_217_ of the age‐matched healthy controls (129.3 fg/mL) was 4.0.

**Conclusion:**

p‐Tau_217_ is a promising blood‐based biomarker in AD research, drug development, diagnosis, disease monitoring, and patient care. The prototype p‐Tau_217_ assay provides fast, highly sensitive, precise, and relatively cost‐effective results in an automated immunoassay on the high‐throughput Beckman Coulter DxI 9000^TM^ Access Immunoassay Analyzer.

